# The Genoprotective Role of Naringin

**DOI:** 10.3390/biom10050700

**Published:** 2020-04-30

**Authors:** Oskar Szczepaniak, Marta Ligaj, Joanna Kobus-Cisowska, Mariusz Tichoniuk, Marcin Dziedziński, Monika Przeor, Piotr Szulc

**Affiliations:** 1 Department of Gastronomy Sciences and Functional Foods, Poznań University of Life Sciences, 60-637 Poznań, Poland; joanna.kobus@up.poznan.pl (J.K.-C.); marcin.dziedzinski@up.poznan.pl (M.D.); monika.przeor@up.poznan.pl (M.P.); 2 Department of Industrial Products Quality and Packaging Development, Poznań University of Economics and Business, 61-875 Poznań, Poland; marta.ligaj@ue.poznan.pl (M.L.); mariusz.tichoniuk@ue.poznan.pl (M.T.); 3 Department of Agronomy, Poznań University of Life Sciences, 60-637 Poznań, Poland; piotr.szulc@up.poznan.pl

**Keywords:** naringin, DNA, reactive oxygen species, electrochemical DNA biosensor, square-wave voltammetry

## Abstract

Since ancient times, fruits and edible plants have played a special role in the human diet for enhancing health and maintaining youthfulness. The aim of our work was to determine the interactions between naringin, a natural ingredient of grapefruits, and DNA using an electrochemical biosensor. Electrochemical methods allow analyzing the damages occurring in the structure of nucleic acids and their interactions with xenobiotics. Our study showed that the changes in the location of electrochemical signals and their intensity resulted from the structural alterations in DNA. The signal of adenine was affected at lower concentrations of naringin, but the signal of guanine was unaffected in the same condition. The dynamics of changes occurring in the peak height and surface of adenine related to naringin concentration was also significantly lower. The complete binding of all adenine bases present in the tested double-stranded DNA solution was observed at naringin concentrations ranging from 8.5 to 10.0 µM. At larger concentrations, this active compound exerted an oxidizing effect on DNA. However, the critical concentrations of naringin were found to be more than twice as high as the dose absorbable in an average human (4 µM). The results of our work might be helpful in the construction of electrochemical sensors for testing the content of polyphenols and would allow determining their genoprotective functionality.

## 1. Introduction

The impact of nutrition on human health has been studied since ancient times. Herbs and various colorful plants have played an important role in the diet of our ancestors. They were considered to have a positive effect on human health and maintain youthfulness [1,2,3]. Today, once again, people pay increasing attention to plants that will help them protect their health. A good example of this trend is the rapid development of nutrigenetics, a discipline that deals with the impact of food and its ingredients on the human genotype [4].

The food components that are widely studied in this regard are flavonoids. These secondary metabolites are responsible, among others, for the flavor, aroma, and color of plants. Recent studies have shown that due to their unique structural properties, flavonoids act as antioxidative and anti-inflammatory agents. Naringenin, a natural ingredient of grapefruits, belongs to the group of flavonoids, and based on its chemical structure, it is known as an aglycone of naringin. Naringin possesses significant antioxidative properties, and its antioxidant activity has been studied in vitro using mouse embryonic fibroblast cell lines (NIH-3T3) exposed to ultraviolet-B radiation [5] and in vivo with insulin-deficient diabetic mice exposed to oxidative stress induced by streptozotocin [6]. The in vitro study showed that naringin prevented ultraviolet-B-mediated inflammation and oxidative damage in the analyzed NIH-3T3 cells by controlling defined inflammatory factors and activating an anti-inflammatory agent present in the cells [5]. The in vivo study (supported by an in vitro analysis) confirmed the multidirectional antioxidant activity of naringin, including the suppression of DNA damage, reduction in the accumulation of reactive oxygen species (ROS) in the pancreas, and protection of the pancreatic β-cells against oxidative stress-induced apoptosis [6].

The aim of our work was to assess the interactions between naringin and nucleic acid using an electrochemical DNA biosensor. Although a similar study has been conducted with cyclic voltammetry [5], no information was provided about the differentiated affinity of naringin to ssDNA and dsDNA. To date, electrochemical methods are considered as a useful tool for the assessment of DNA damages and the interaction of nucleic acid with xenobiotics [6]. The observation of shifts in the electric potential of the signals of DNA associated with its structural changes has confirmed that the oxidized forms of nucleobases have other oxidation potentials [7,8]. A substance interacting with DNA may exhibit various strengths of interaction depending on its concentration, which also results in changes in the area and intensity of the measured electrochemical signals. For instance, a protective effect induced by the interaction may lead to a decrease in the area and intensity of the observed DNA signals [9]. In the present study, such electrochemical changes were analyzed to investigate the interactions between a DNA sample and naringin solution at different concentrations.

We proposed the following two research hypotheses.

**Hypothesis** **1.** *Naringin interacts with oligonucleotide and double-stranded DNA, protecting it against oxidation.*

**Hypothesis** **2.** *The higher concentration of naringin in the DNA solution, the more significant protection before DNA oxidation.*

The strength of xenobiotics binding to a minor groove depends mainly on the pair of nucleobases located in the groove and the optimal ratio of the macromolecules and nucleotide. Crystallographic studies have shown that the most stable hydrogen bonds are obtained for adenine-thymine pair at a molecular ratio of 1:1 of the pair and xenobiotic [10]. In the case of guanine-cytosine pair, two molecules are needed, both with aromatic rings located one above other. Naringin may act similarly as it contains a large planar sugar ring. Mountzouris and Hurley [10] noted that numerous polycyclic compounds exhibited such activity due to steric hindrance; for example, in the G-C pair, this activity is caused by a primary amine group. Another key factor for this activity is the charge distribution in the range of two base pairs. G-C pair is less nucleophilic than A-T, which reduces the feasibility of creating a stable hydrogen bond.

Therefore, we proposed the following third hypothesis.

**Hypothesis** **3.** *Naringin would bind less strongly to the G-C base pair than the A-T base pair.*

The findings of our study might help in the construction of biosensor, assessing the pro-health potential of tested fruits extracts, juices, and functional drinks. They also could be a good base for future research concerning DNA-phytochemicals interactions.

## 2. Materials and Methods

### 2.1. Materials

In the study, we used an HPLC-grade standard of naringin (Merck, Darmstadt, Germany), 0.05 M phosphate buffer added with 0.01 M KCl (pH 7.0), and pure water. Carbon paste for the working electrode (CPE) was made using graphite powder (Sigma-Aldrich, Steinheim, Germany) and mineral oil (Sigma-Aldrich, Steinheim, Germany), mixed at the ratio of 7:3 (*w*/*w*) [11]. A platinum wire was used as an auxiliary electrode, and Ag/AgCl (3 M KCl) electrode was applied as a reference.

We used two types of nucleic acid samples: a synthetic oligonucleotide with the sequence 5′GCTCGGTACGGAAGTTGAC (Tib Molbiol, Poznań, Poland) and a double-stranded calf thymus DNA (D-1501, Sigma-Aldrich, Steinheim, Germany) dissolved in phosphate buffer.

### 2.2. Methods

**Figure A1:**
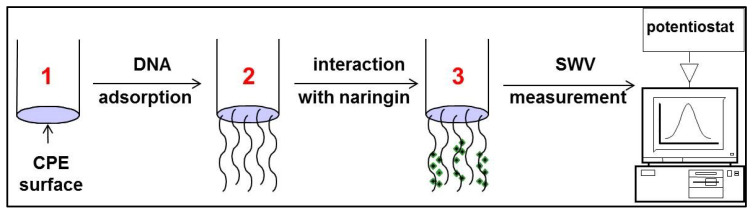
Graphic illustration of SWV methodology and measurement schemes.

A graphical illustration of the applied methods is presented in Figure A1.

#### 2.2.1. Naringin Electrochemical Response

Seven standard aqueous solutions of naringin were prepared at the following concentrations: 2.5, 5.0, 10.0, 20.0, 30.0, 40.0, and 50.0 μM. A 100 μL of each test solution was added to 900 μL of phosphate buffer. In the first step, the electrodes were placed in a well filled with 1 mL of phosphate buffer. After 60 s of conditioning at a potential of +1.7 V and 5 s of equilibration, a background signal was measured at a range of 0.01–1.40 V with an amplitude of 0.04 V and a frequency of 50 Hz. The calibration curve was smoothed using the Savitzky-Golay algorithm. Then, the electrode set was transferred to the naringin solution. Naringin was deposited on the surface of CPE at a potential of +0.5 V for 120 s. The electrodes were subsequently washed in a fresh buffer for 30 s, and a square-wave voltammetric (SWV) measurement was carried out. The obtained voltamperogram was smoothed, and then a background baseline was subtracted from it. The prepared voltamperogram was used in the statistical analysis for determining the SWV peak area. During the stages of conditioning, depositing, and washing, the solutions were stirred at a rate of 200 rpm. The carbon paste from the surface of the working electrode was manually rubbed off using a filter paper after each SWV measurement, a new layer of fresh paste was covered, manually moved, and smoothed using a matt microscope slide (Figure A1, Stage 1).

Linear curves were constructed based on the individual peak area of naringin and the total area to select the best linear model.

#### 2.2.2. Interaction of Naringin with Synthetic Oligonucleotide and dsDNA

The procedure was conducted following the method of Jasnowska et al. [12]. The SWV measurement started with the control measurement of the buffer background. Electrodes were placed in a well filled with 1 mL of phosphate buffer. After 60 s of conditioning, the electrode set in the buffer solution (at a potential of +1.7 V and 5 s of equilibration time), and a calibration signal was measured at a range of 0.01–1.40 V with an amplitude of 0.04 V and a frequency of 50 Hz. The calibration curve was smoothed using the Savitzky-Golay algorithm. Then, the electrode set was transferred to either 10 μM oligonucleotide solution or 20 μg/mL double-stranded DNA (dsDNA) solution (Figure A1, Stage 2). The nucleic acid was deposited on the surface of CPE charged to a potential of +0.5 V for 120 s. The electrode set was subsequently moved to a naringin buffer solution (Figure A1, Stage 3), in which a no-current deposition of naringin was conducted for only 60 s, as a long time could cause desorption of the bounded DNA. Then, the electrode set was washed in a fresh buffer for 30 s, and the SWV measurement was carried out. The obtained voltamperogram was smoothed, and then a background baseline was subtracted from it. The corrected voltamperogram was saved, and the peak area was statistically analyzed. During the stages of conditioning, depositing, and washing, the solutions were stirred at a rate of 200 rpm. After each measurement, the layer of CPE was regenerated according to the procedure described above.

As a blank sample, a signal of oligonucleotide or dsDNA was measured without naringin deposition.

#### 2.2.3. Statistical Analysis

Statistical analysis was performed using Origin Pro ver. 7.0 software (OriginLab, Northampton, MA, USA). The relative standard deviation (RSD) was calculated for each sample. A linear response model with an analysis of variance (ANOVA) was used for determination with respect to naringin concentration. The relationship between the individual peaks of oligonucleotide and naringin solutions was analyzed based on the Boltzmann equation.

(1) $$y=\frac{A_{1}-A_{2}}{1+e^{\frac{x-x_{0}}{dx}}}+A_{2}$$

where *A*_1_, *A*_2_, *x*_0_, and *dx* are Constants.

The statistical relationship between the individual peak area of dsDNA and naringin concentration was analyzed using the polynomial regression model, described in Equation (2).

y = Ax^3^ + Bx^2^ + Cx + D (2)

where A, B, C, and D are constants.

Relative peak area and relative shift were calculated using the following equations:

(3) $$Relative\;peak\;area=\frac{\left[\left(peak\;area\;after\;interaction\right)-\left(peak\;area\;before\;interaction\right)\right]}{\left(peak\;area\;before\;interaction\right)}*100\;\%$$

(4) $$Relative\;shift=\frac{\left[\left(peak\;location\;after\;interaction\right)-\left(peak\;location\;before\;interaction\right)\right]}{\left(peak\;location\;before\;interaction\right)}*100\;\%$$

## 3. Results

### 3.1. Electrochemical (SWV) Signals of Naringin

#### 3.1.1. Naringin Electrochemical Signals

**Figure 1:**
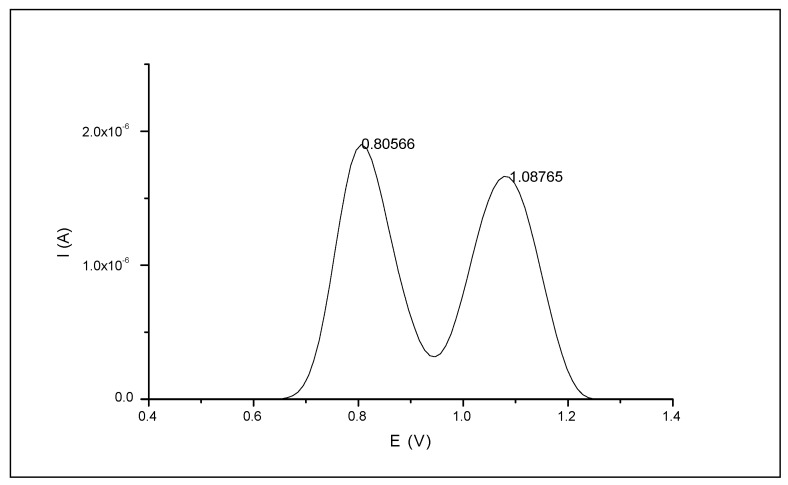
Square-wave voltamperogram of 50 µM naringin solution.

The SWV voltamperogram of 50 µM naringin showed two clear peaks located at the potential values of +0.8 and +1.1 V, respectively (Figure 1). In the next steps, the measurements were conducted with lower concentrations of the target compound.

**Figure A2:**
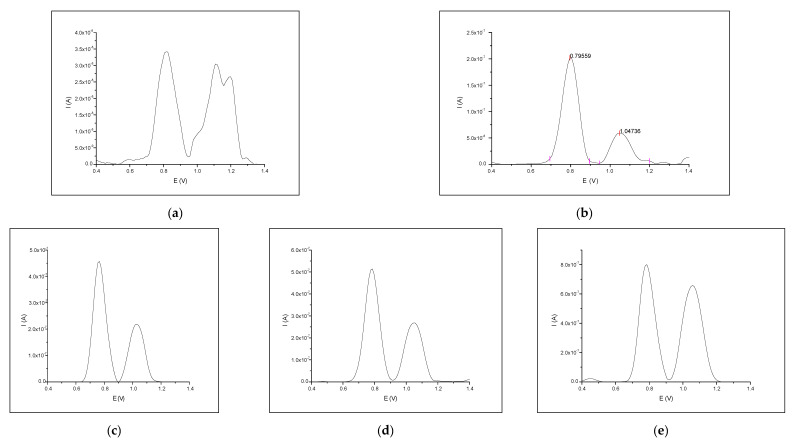
Average voltamperograms of different concentrations of naringin: (**a**) 0.5 μM; (**b**) 2.5 μM; (**c**) 5.0 μM; (**d**) 10.0 μM; (**e**) 20 μM.

The obtained SWV voltamperograms (Figure A2) confirmed that the SWV signal of naringin was detectable even at a concentration of 0.5 µM (the registered peak had a current value of 10^−8^ A). Moreover, it could be observed that the higher the analyte concentration, the higher the value, which was revealed by the peak located beyond the potential of +1.1 V in relation to the peak at +0.8 V.

#### 3.1.2. Calibration Curve

The areas of both the SWV peaks of naringin were used in the analysis to study the electrochemical response of this target compound to the increase in its concentration. Totally, three calibration curves were plotted: first one for the area of peak at +0.8 V (S1), second for the peak area at +1.0 V (S2), and the last for the total peak area (SN). Table 1 shows the linearity parameters of the curves and ANOVA test results.

Calibration curves plotted for each individual peak area (S1 and S2, respectively) had excellent regression parameters. Their *r*^2^ values were similar, and both were applied in further analyses. Linearity calculated for the S2 electrochemical response of naringin had the smallest error compared to the other two described models.

However, we used the SN calibration model in further analyses because it had the highest *r*^2^ parameter, and its *F* value was close to that of the S2 model.

### 3.2. Interaction between Naringin and Oligonucleotide

**Figure 2:**
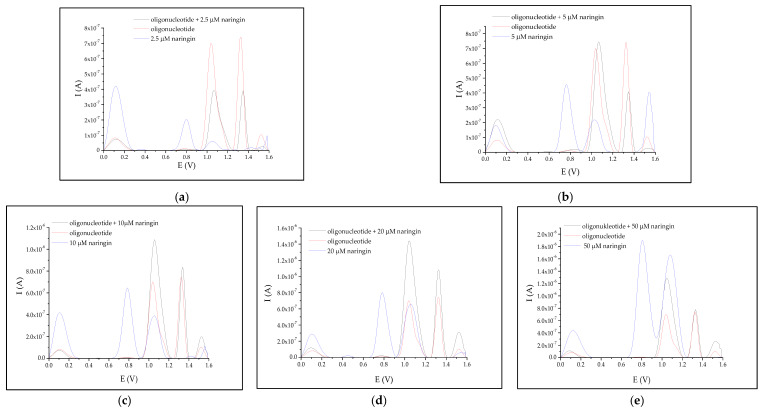
Average voltamperograms of tested oligonucleotide before (red line) and after (black line) interaction with growing concentrations of naringin (**a**–**e**).

**Figure A3:**
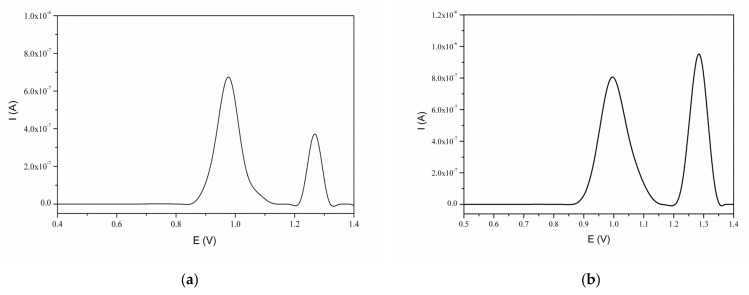
Average electrochemical signal of oligonucleotide: 5′GCTCGGTACGGAAGTTGAC (**a**) and calf thymus DNA (**b**).

SWV voltamperogram of the analyzed oligonucleotide showed two peaks: the first one with the maximum at +1.04 V, and the second with the maximum at +1.33 V (Figure A3). Figure 2 presents the voltamperograms obtained for the oligonucleotide before (red line) and after (black line) treatment with naringin at different concentrations (ranging from 2.5 to 50.0 µM). In Figure 2, voltammograms obtained for oligonucleotide and oligonucleotide after interaction with naringin at tested concentrations are present. For comparison, the naringin signal at the same concentration was also included.

**Remark** **1.** *The signal of pure naringin measured on the bare carbon paste electrode had no reference to the measurement performed on the electrode coated with the nucleic acids’ layer.*

In the first situation, adsorption of naringin in the carbon paste occurred under the influence of the applied potential; in the second case, the surface of the electrode was covered with a layer of DNA, and after 60 s of interaction with naringin (without applied potential) and 30 s of rinsing to remove unbound naringin, we observed the effect of this interaction.

**Proof of Hypotheses 1 and 2.**  It was apparent that an increase in naringin concentration led to an increase in the intensity of the SWV peak located at the potential of +1.0 V. □

However, the 2.5 µM concentration of naringin resulted in a slightly lower peak intensity (Figure 2a). The intensity of the latter signal of the oligonucleotide increased simultaneously with the increase in naringin concentration, but at the concentration range of 2.5–5 μM, the peak intensity was lower than that observed before interaction. Although the two-fold increase in naringin concentration resulted in a nearly 200% gain of the first electrochemical signal of the oligonucleotide, the intensity of the latter single-stranded DNA (ssDNA) signal increased only by 100% (Figure 2c).

**Proof of Hypothesis 1.**  By observing all the measured SWV voltamperograms, it was clear that the interaction of nucleic acids with naringin in the tested solutions shifted the maxima of oligonucleotide peaks toward higher potential values; for example, the maxima of the first peak shifted from +1.037 to +1.067 V (Table 2). □

However, the dynamics of these shifts was insignificant.

The naringin concentration corresponded to the peak area located at about +1.3 V, which remained unchanged after the interaction of DNA with the flavonoid, at a concentration of 10 µM (Table 2). The initial naringin concentration that corresponded to the +1.0 V peak was about 3.0 µM and fluctuated between 2.5 and 5.

### 3.3. Interaction of Naringin with dsDNA

Calf thymus DNA was used as a model of dsDNA. This nucleic acid sample allowed achieving highly repeatable SWV responses in the form of two electrochemical signals located at a potential range similar to those obtained for the oligonucleotide analyzed before (Figure A3).

**Figure 3:**
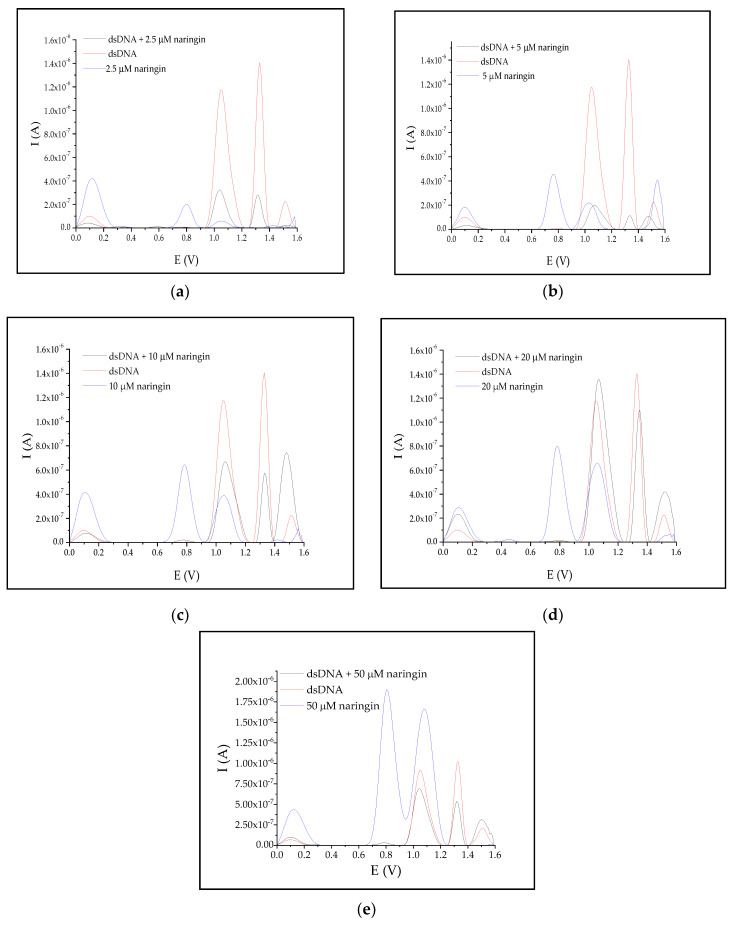
SWV voltamperograms of dsDNA before (red line) and after (black line) interaction with growing concentrations of naringin (**a**–**e**).

**Proof of Hypothesis 2.**  The interaction study showed that each tested solution of naringin at a concentration below 20 μM gave rise to the SWV peak at 1.0 V that was lower in intensity compared to the peak obtained before DNA interaction with the analyte (Figure 3). The intensity of the latter peak (+1.3 V) decreased in all samples, except for naringin concentration of 30 and 40 μM, after interaction in comparison with the initial electrochemical signal of DNA. □

**Proof of Hypothesis 3.**  The naringin solution that resulted in no changes in the intensity of 1.0 V peak after interaction had a concentration of 16 µM (Table 3). The concentration values for the other peak not associated with any electrochemical signal changes were determined by the extrapolation of the data from Table 3 using a model from Table 4. The calculated concentration value was 26 µM. □

**Proof of Hypothesis 2.**  A significant drop in electrochemical response could be observed for both the peak areas of dsDNA after the interaction of DNA with the naringin solution at a concentration of 5 μM (Table 3). □

The changes in the areas and location of both peaks revealed that the analyzed range of naringin concentration could be divided into two subgroups: 2.5 and 5 μM, for which the areas of both peaks declined, while the latter higher concentration resulted in an increase in the area of the first peak after DNA interaction with naringin. The shifts in peak potentials seemed to be occurring randomly in the whole tested range of concentration. A significantly higher peak area was obtained over 20 μM concentration of naringin, reached the highest value for 30 μM, and it was associated with a noticeable shift of the SWV signal located at +1.3 V toward a more positive potential value. An increase of the latter peak area was observed after interaction with naringin concentration 30 and 40 μM, whose signal had also been slightly shifted towards higher potential.

The conclusion derived from the analytical data presented above was supported by the regression and statistical analyses, which illustrated a statistical relationship between the naringin concentration and the areas of the recorded electrochemical signals of DNA.

### 3.4. Correlation Analyses

**Figure 4:**
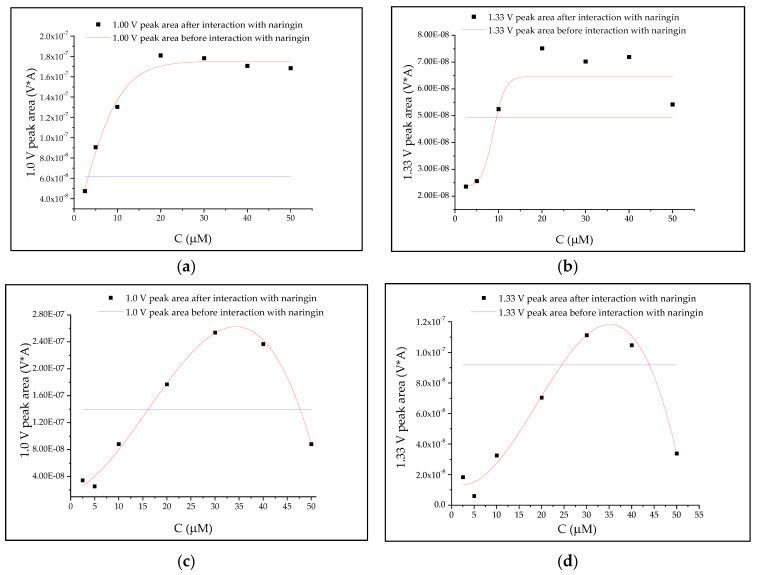
The relation between the areas of oligonucleotide (**a**,**b**) and dsDNA (**c**,**d**) peaks at +1.0 V (**a**,**c**) and +1.3 V (**b**,**d**) and the concentration of naringin.

A Boltzmann regression model was found to be the most suitable for studying the naringin interactions with an oligonucleotide (Figure 4a,b), while for analyzing the interactions with dsDNA, a polynomial regression model was useful (Figure 4c,d). The results of the statistical analyses proved that interaction with naringin at low concentration caused a decline in the area of both peaks in the case of tested nucleic acids’ samples, while the increasing naringin concentration led to an increase in the peak areas. The naringin concentration that caused no change in the area was found to be higher for the SWV peak recorded at +1.3 V than the concentration related to the signal at +1.0 V.

Boltzmann correlation warranted a meaningful but insignificant relationship between the peak area of oligonucleotide and naringin concentrations (Table 4). Surprisingly, the model that was based on the areas of the SWV peak located at lower potential (the first one) was found to be more precise.

Similar results were observed in the study of the naringin interaction with dsDNA. The correlation between the first peak area and naringin concentration was also much more significant.

## 4. Discussion

The influence of naringin at low concentrations on both the tested DNA samples caused a drop in the electrochemical signals. This decline was related to the interaction between nucleic acid and xenobiotic, which limited the spatial accessibility of the nucleic acid to the surface of the working electrode [12]. A similar study was performed by Maatouk et al. [5], in which protective action of naringin occurred at the concentration ranging from 0.1 nM to 0.1 μM. The study revealed that the protective effect of naringin might be obtained at an even 50-fold higher dose. The affinity of naringin to ssDNA was also examined.

The mechanism of the SWV analysis of DNA is based on π-π electrons stacking between nitrogenous bases of the nucleic acid and the CPE layer. This mechanism is also applied in the nanocarbon-assisted fluorescence method of nucleic acid analysis and its interaction studies [13]. Moreover, electrochemical methods have been recently considered as rapid and reliable tools for measuring antioxidant capacity and toxicological studies in food matrices [14,15]. During the electrochemical measurement, DNA strands are immobilized on the working electrode surface and intercalating them, xenobiotic (e.g., naringin) possesses higher resistance to oxidation than DNA before interaction [16].

Electrochemical detection of naringin is based on the electrochemical activity of its aromatic rings, just like DNA nucleobases. Naringin as compounds has three aromatic rings, which provide two electrochemical signals. The lower number of registered peaks is an effect of the delocalization of electron charge between two adjacent naringin rings [17].

The registered SWV voltammograms of oligonucleotide had two clear peaks, specific for guanine and adenine. Guanine peak had its maximum near +1.0 V, while the adenine peak was located at approximately +1.3 V [12,18,19]. The RSD calculated for the results was higher than the recommended 10% value, but it could have been caused by random factors, prevalently resulting from difficulties in repetitive regeneration of the CPE layer, which needs a highly precise, almost half-automatic procedure. Moreover, nucleic acids can bind to the detection layer of CPE in an unpredictable way, which may further affect the intensity of signals of individual nucleobases [20,21]. Therefore, numerous measurements of a single sample are carried out, and conclusions are drawn from the average results.

The increase in the SWV signal of adenine (+1.3 V) observed after the interaction of the nucleic acid with naringin was the feasible effect of the growth of guanine signal (+1.0 V), which interacted with the working electrode and naringin at the same time, and could affect the cleavage of the single helical form of the nucleic acid and its accessibility to CPE. In addition, naringin-adenine interactions could cause the ssDNA structure to unravel, resulting in an increase in the intensity of all nucleobases signals. Therefore, to draw a conclusion about the strength of naringin interaction, the collected data were compared to those obtained for the guanine and adenine peaks before interaction (shown in Figure 2 and Figure 3 with red lines). Analyzing the differences between these parameters with respect to the increase in naringin concentrations, we noted the following.

**Proof of Hypothesis 3.**  In line with hypothesis 3, naringin bound more strongly to A-T base pairs and protected it more effectively against electrochemical oxidation compared to G-C pairs. This was visible on the voltammograms as a greater reduction of adenine signal compared to guanine after interaction with all of the tested naringin concentrations (Figure 3). The intensity of the adenine peak increased only by 100% for the same increase in naringin concentration. Naringin could bind with many guanine bases simultaneously, which could be theoretically feasible in the case of dsDNA, where the minor grooves are made of short repetitive guanosine fragments [22,23]. □

**Remark 2.**  *The electrochemical signal of naringin had no significant effect on the electrochemical response of adenine.*

Models based on Boltzmann distribution indicate that above a specific concentration, naringin shows no antioxidative action on ssDNA. Probably, at high doses of naringin, oxidation of DNA might occur, which may be induced by the high concentration of this flavonoid [24,25,26]. Large quantities of naringin can cause breakage and cleavage of genetic material into less fragments, as ssDNA is surrounded by a sufficient number of naringin molecules placed across the deoxyribose-phosphate chain. Such cleavage would result in oligonucleotide decay into smaller fragments, as numerous studies have reported [24,25,26]. However, this phenomenon is connected with the high reactivity of the superoxide form of naringenin, which is obtained as a product of the reaction between naringin and ROS. Nevertheless, DNA oxidation would be seen in voltamperograms as, *inter alia*, the growing 8-oxoguanine peak at +0.8 V [9]. Such peaks were noticeable for both oligonucleotide and dsDNA (Figure 2 and Figure 3), but the dynamics of their accumulation seemed to be negligible. In the serum of healthy human cells, the biological absorption of naringin is limited to less than 4 μM [27]. Therefore, a clear antioxidant effect could only be observed in vivo.

Figure 4c shows that for tested naringin solutions from 2.5 to 10 μM, the degree of changes in the area of guanine peak was less than 100%. The concentration of naringin that did not affect the guanine peak was 16 µM (Figure 4c). The previous research demonstrated that the binding constant between ctDNA and naringin equaled 4.7 × 10^4^ M^−1^ using the absorbance method and 8.22 × 10^4^ M^−1^ with the application of fluorescence one [28]. However, the authors of the study did not test differences in binding capacity between A-T and G-C base pairs.

In the case of adenine (Figure 4d), only the tested naringin solutions at a concentration of 30 and 40 μM increased the signal surface area of the nucleobase in comparison to the dsDNA signal before the interaction. In the in vitro and in silico study performed by Yousouf and Enoch [28], naringin bound to ctDNA at stoichiometric ratio 1:1.5. It was clearly seen that in dsDNA, adenine was more protected against oxidation than guanine, for which the drop in the peak area was stronger for each tested concentration. Bhattacharjee et al. [26] confirmed that naringin-DNA interactions were long-range ones, which included hydrogen bonding. The strength of the interaction is limited by the presence of neohesperidoside ligand [28]. In the cited study, the predicted O–H hydrogen bonds ranged between 1.6 and 2.3 Å. The interactions are stronger when the differences in the nucleophilic properties between naringenin and the nucleobase are larger. Therefore, naringenin binds strongly to adenine than to guanine. This is in contrast to Mello et al.’s study, who showed that the Cu-naringin complex had a greater affinity to interact with guanine [29]. It may be explained by different electron charge delocalization in this complex and its electroactivity.

**Remark 3.**  *This indicated that naringin exerted a better antioxidative effect on dsDNA than ssDNA.*

The larger concentration of naringin was associated with a higher probability of interaction with dsDNA. Moreover, Hoogsteen-type bonds may induce an increase in internal energy and changes in complex geometry [30,31].

**Remark 4.**  *A double-helical DNA structure was more preferable to naringin than ssDNA.*

The above remark was derived from a comparison between naringin concentrations that did not cause an increase in the intensity of naringin with guanine derived from ssDNA, while the highest strength was found for interaction of naringin with adenine from dsDNA (Figure 4).

## 5. Conclusions

Naringin exhibited a significant antioxidant effect at a concentration of up to 10 μM. Above this level, this xenobiotic caused structural deformations in the tested nucleic acids, which did not allow drawing clear conclusions and indicated that an excessive amount of naringin might even induce DNA oxidation. Although naringin bound to guanine with a two-fold higher strength than to adenine, the interaction with the latter one resulted in much better antioxidative protection. The DNA structure also implied the genoprotective properties, and, especially, the double-helical structure seemed to be more favorable.

## Figures and Tables

**Table 1:** Statistical parameters of three calibration curves based on naringin peak areas.

Calibration Curve	Intercept		Slope				*r* ^2^
			Value		Standard Deviation		
**SN**	0		1.014 × 10^−8^		0.029 × 10^−8^		0.99495
	**Variance**	**Degrees of freedom**	**Total squares**	**Mean squares**		***F* value**	***p* > F**
	Between samples	1	3.117 × 10^−13^	3.117 × 10^−13^		1184.254	3.897 × 10^−7^
	Inside sample	5	1.316 × 10^−15^	0.263 × 10^−15^			
	Sum of totals	6	3.130 × 10^−13^				
**S1**	**Intercept**		**Slope**				***r*^2^**
			Value		Standard deviation		
	0		5.116 × 10^−9^		0.228 × 10^−9^		0.98816
	**Variance**	**Degrees of freedom**	**Total squares**	**Mean squares**		***F* value**	***p* > F**
	Between samples	1	7.934 × 10^−14^	7.934 × 10^−14^		501.800	3.294 × 10^−6^
	Inside sample	5	7.906 × 10^−16^	1.581 × 10^−16^			
	Sum of totals	6	8.013 × 10^−14^				
**S2**	**Intercept**		**Slope**				***r*^2^**
			Value		Standard deviation		
	0		5.023 × 10^−9^		0.162 × 10^−9^		0.99379
	**Variance**	**Degrees of freedom**	**Total squares**	**Mean squares**		***F* value**	***p* > F**
	Between samples	1	7.649 × 10^−14^	7.649 × 10^−14^		961.392	6.550 × 10^−7^
	Inside sample	5	3.978 × 10^−16^	0.796 × 10^−16^			
	Sum of totals	6	7.689 × 10^−14^				

S1—area of the 0.8 V peak; S2—area of 1.0 V peak; SN—total area of both peaks.

**Table 2:** Electrochemical parameters describing the naringin-oligonucleotide interaction.

Naringin Concentration (μM)	N	Average Peak Area (A × V)				Relative Peak Area (%)	
		+1.0 V after Interaction	+1.3 V after Interaction	+1.0 V before Interaction	+1.3 V before Interaction	+1.0 V after Interaction	+1.3 V after Interaction
2.5	5	4.762 × 10^−8^	2.358 × 10^−8^	6.154 × 10^−8^	4.935 × 10^−8^	77.38	47.79
5.0	4	9.075 × 10^−8^	2.565 × 10^−8^			147.46	51.97
10.0	3	1.304 × 10^−7^	5.248 × 10^−8^			211.91	106.34
20.0	4	1.810 × 10^−7^	7.518 × 10^−8^			294.12	152.33
30.0	3	1.783 × 10^−7^	7.025 × 10^−8^			289.73	142.35
40.0	2	1.707 × 10^−7^	7.191 × 10^−8^			277.38	145.71
50.0	2	1.685 × 10^−7^	5.424 × 10^−8^			273.73	109.90
**Naringin Concentration (μM)**	**N**	**First Peak Maximum Location (V)**		**Latter Peak Maximum Location (V)**		**Relative Shift (%)**	
		**After Interaction**	**Before Interaction**	**After Interaction**	**Before Interaction**	**First Peak**	**Latter Peak**
2.5	5	1.067	1.037	1.349	1.329	2.89	1.50
5.0	4	1.067		1.349		2.89	1.50
10.0	3	1.057		1.339		1.93	0.75
20.0	4	1.047		1.329		0.96	0
30.0	3	1.047		1.329		0.96	0
40.0	3	1.047		1.329		0.96	0
50.0	2	1.047		1.329		0.96	0

**Table 3:** Electrochemical parameters describing the naringin-dsDNA interaction.

Naringin Concentration (μM)	N	Average Peak Area				Relative Peak Area	
		V × A				%	
		+1.0 V after Interaction	+1.3 V after Interaction	+1.0 V before Interaction	+1.3 V before Interaction	+1.0 V after Interaction	+1.3 V after Interaction
2.5	3	3.445 × 10^−8^	1.836 × 10^−8^	1.394 × 10^−7^	9.179 × 10^−8^	24.72	20.00
5.0	4	2.557 × 10^−8^	6.033 × 10−^9^			18.34	6.57
10.0	3	8.825 × 10^−8^	3.253 × 10^−8^			63.31	35.44
20.0	4	1.769 × 10^−7^	7.043 × 10^−8^			126.87	76.73
30.0	3	2.538 × 10^−7^	1.112 × 10^−7^			182.07	121.15
40.0	3	2.367 × 10^−7^	1.047 × 10^−7^			169.80	114.06
50.0	5	8.808 × 10^−8^	3.385 × 10^−8^			63.19	36.88
**Naringin concentration** **(μM)**	**N**	**First peak maximum location (V)**		**Latter peak maximum location (V)**		**Relative shift (%)**	
		**After interaction**	**Before interaction**	**After interaction**	**Before interaction**	**First peak**	**Latter peak**
2.5	3	1.037	1.047	1.319	1.329	−0.96	−0.75
5.0	4	1.067		1.339		1.91	0.75
10.0	3	1.067		1.329		1.91	0
20.0	4	1.067		1.349		1.91	1.50
30.0	3	1.067		1.339		1.91	0.75
40.0	3	1.067		1.339		1.91	0.75
50.0	5	1.047		1.319		0	−0.75

**Table 4:** Correlation equation parameters and statistics between oligonucleotide/dsDNA individual peak areas and naringin concentration.

Oligonucleotide						
Peak Area	A1	A2	x_0_	dx	Statistics	
					*Χ* ^2^	*r* ^2^
+1.0 V	−1.126 × 10^−7^	1.754 × 10^−7^	1.330	4.621	2.345 × 10^−16^	0.9232
+1.3 V	2.334 × 10^−8^	6.470 × 10^−8^	8.839	1.32944	2.195 × 10^−16^	0.5324
**dsDNA**						
Peak area	A	B	C	D	Statistics	
					*Χ* ^2^	*r* ^2^
+1.0 V	1.750 × 10^−8^	2.423 × 10^−9^	4.822 × 10^−10^	−1.005 × 10^−11^	3.573 × 10^−16^	0.9015
+1.3 V	1.541 × 10^−8^	−1.606 × 10^−9^	3.408 × 10^−10^	−6.027 × 10^−12^	1.346 × 10^−16^	0.7700

A_1_, A_2_, x_0_, and dx are Boltzmann parameter constants described in Section 2.2.3; A, B, C, and D are constants of the polynomial equation described in Section 2.2.3.
